# Long term culture of mesenchymal stem cells in hypoxia promotes a genetic program maintaining their undifferentiated and multipotent status

**DOI:** 10.1186/1471-2121-12-12

**Published:** 2011-03-30

**Authors:** Leticia Basciano, Christophe Nemos, Bernard Foliguet, Natalia de Isla, Marcelo de Carvalho, Nguyen Tran, Ali Dalloul

**Affiliations:** 1 Nancy University Medical School (EA 4369) and School of Surgery (NT), 54500 Vandœuvre-lès-Nancy, France

## Abstract

**Background:**

In the bone marrow, hematopietic and mesenchymal stem cells form a unique niche in which the oxygen tension is low. Hypoxia may have a role in maintaining stem cell fate, self renewal and multipotency. However, whereas most studies addressed the effect of transient *in vitro *exposure of MSC to hypoxia, permanent culture under hypoxia should reflect the better physiological conditions.

**Results:**

Morphologic studies, differentiation and transcriptional profiling experiments were performed on MSC cultured in normoxia (21% O_2_) versus hypoxia (5% O_2_) for up to passage 2. Cells at passage 0 and at passage 2 were compared, and those at passage 0 in hypoxia generated fewer and smaller colonies than in normoxia. In parallel, MSC displayed (>4 fold) inhibition of genes involved in DNA metabolism, cell cycle progression and chromosome cohesion whereas transcripts involved in adhesion and metabolism (CD93, ESAM, VWF, PLVAP, ANGPT2, LEP, TCF1) were stimulated. Compared to normoxic cells, hypoxic cells were morphologically undifferentiated and contained less mitochondrias. After this lag phase, cells at passage 2 in hypoxia outgrew the cells cultured in normoxia and displayed an enhanced expression of genes (4-60 fold) involved in extracellular matrix assembly (SMOC2), neural and muscle development (NOG, GPR56, SNTG2, LAMA) and epithelial development (DMKN). This group described herein for the first time was assigned by the Gene Ontology program to "plasticity".

**Conclusion:**

The duration of hypoxemia is a critical parameter in the differentiation capacity of MSC. Even in growth promoting conditions, hypoxia enhanced a genetic program that maintained the cells undifferentiated and multipotent. This condition may better reflect the *in vivo *gene signature of MSC, with potential implications in regenerative medicine.

## Background

Adult bone marrow is a widely used source of mesenchymal stem cells (MSC) that can be isolated and expanded in culture while keeping the ability to form adipocytes, chondrocytes and osteoblasts [1,2] and possibly other cell types including cardiomyocytes [3]. Within the bone marrow, MSC may interact with hemopoietic stem cells (HSC), which reside in a specific microenvironment formed by various stromal precursor cells and osteoblasts, called the niche [4-6]. Whether MSC reside in the same niche amidst HSC or whether they dwell in a specific niche is presently unknown. Different types of niches for hemopoietic progenitors may exist depending on their more or less primitive state [7] located near bone surfaces away from blood vessels and therefore submitted to a low O_2 _tension. It is thus inferred that stem cells are equipped to survive in a hypoxic environment and that this condition plays a role in the maintenance of multipotency [8] and extension of survival [9]. This may hold true for murine and human MSC as their proliferation, differentiation and survival [10-12] are affected by culture in low O_2 _tension. However the degree and duration of hypoxia described in the literature vary greatly and may result in opposite effects on the proliferation and differentiation capacities of MSC [13-15]. So far one study described the long term (one month) effect of human MSC culture under low O_2 _tension (2% O_2_) and showed improved survival and increase in adipocytic and osteogenic differentiation capacity [16]. In the present study we cultured human MSC in normoxia (21% O_2_) versus hypoxia (5% O_2_) for up to passage 3 (P3) and compared their morphology differentiation potential and mRNA expression at early and late passages. We observed that cells cultured under low O_2 _tension were more undifferentiated than cells cultured in normoxia. Further, hypoxia inhibited the expression of genes involved in DNA replication and cell division at P0. At P2, however, Gene Ontology (GO) analysis revealed that only one significant functional group of genes was stimulated and related to "plasticity". We conclude that culture in hypoxia maintains MSC in a multipotent, undifferentiated state.

## Results

### The effect of hypoxia on MSC expansion and phenotype

**Figure 1:**
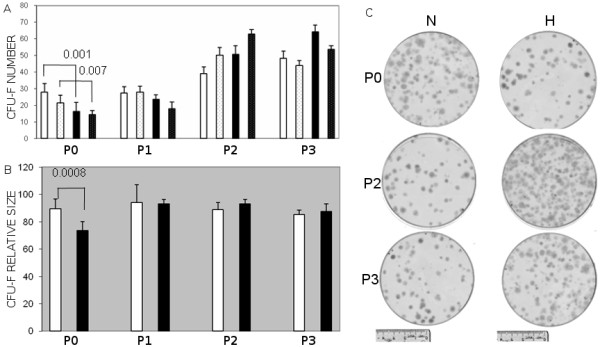
**The effect of oxygen tension on CFU-F size and numbers**. Cells were plated at 1000 (plain histograms) and 10000 MNC/60 cm^2 ^(dotted histograms) from total BM for P0. For the next passages, 100 (plain histograms) and 1000 cells/cm^2 ^(dotted histograms) were seeded. Cells were incubated under hypoxic (5% O_2_, black histograms) or normoxic conditions (21% O_2_, white histograms) respectively and colonies were counted and their size evaluated (A & B). Mean +/- of 3 to 5 independent experiments. Numbers above the histograms (A & B) represent the significance calculated using bilateral paired Student's t test. A representative aspect of colonies at various passages is shown (C).

Bone marrow mononuclear cells (MNC) were cultured and passaged until P3. As shown in Figure 1, both the CFU-F numbers and the mean colony size were significantly smaller at P0 in 5% O_2 _(hypoxia) versus 21% O_2 _(normoxia). This diminution was however less significant at P1 (0.05<p < 0.1), and at P2 the numbers of CFU-F were enhanced by hypoxia. In other experiments, cells were trypsined and counted, total cell numbers were diminished in hypoxia versus normoxia at P0 whereas they were enhanced at P1; the overall cell doubling/day was diminished by hypoxia until P1 and augmented afterwards (data not shown).

**Figure 2:**
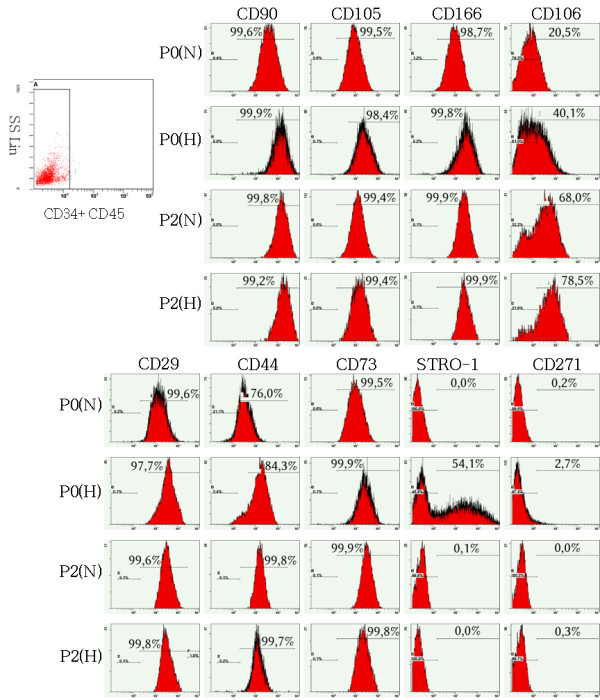
**Stability of MSC phenotype in culture**. Flow cytometry analysis of surface markers at P0 and P2 in hypoxia and normoxia. Histograms show the intensity of several markers within the CD34-CD45- (dot plot), MSC-enriched population from the bone marrow. A representative experiment out of 3 from distinct donors is shown.

In parallel, immunostaining and flow cytometry were performed at various time points. MSC were negative for CD45 and CD34 and positive albeit variably for several other markers (Figure 2). In brief, cultured cells displayed a typical MSC profile with stable phenotype overtime and no significant phenotypic differences between hypoxic and normoxic conditions in agreement with others [17]. Only STRO-1 was transiently expressed on 50% of the cells at P0 under hypoxia and diminished thereafter as expected from previous observations [18]. In brief, after a lag phase during which the hypoxic cells grew slower than normoxic cells, the former expanded faster in late passages. In contrast, with the exception of STRO-1 which is dicussed in the relevant section, the phenotype of MSC was not modified during culture expansion irrespective of the oxygen tension. We next looked for qualitative effects of hypoxia and investigated the morphology of MSC by light and electron microscopy and evaluated the numbers of mitochondria in hypoxic versus normoxic cells.

### Culture of MSC in hypoxia inhibited cell differentiation and mitochondrial biogenesis

**Figure 3:**
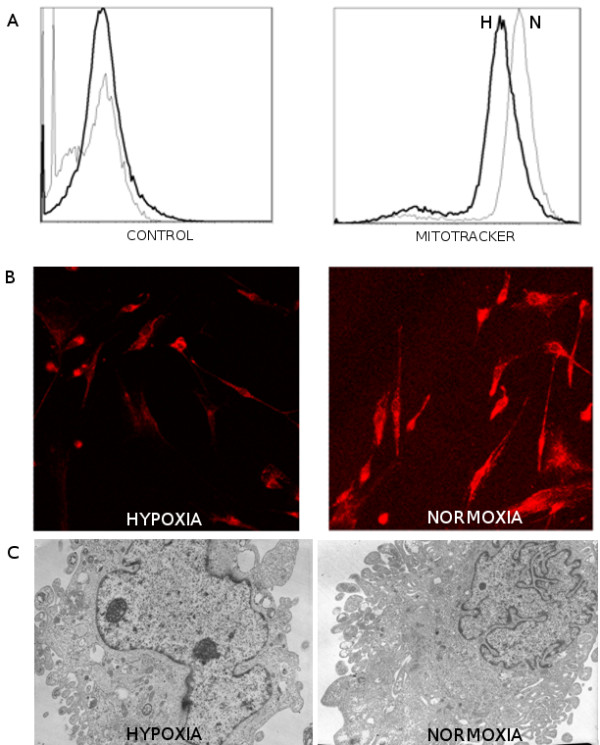
**Morphological aspect of MSC**. The amount of mitochondria was evaluated by flow cytometry (A) and optical microscopy (B) on MSC stained with Mitotracker Orange. TEM was performed on MSC at P2 culture in normoxic or hypoxic conditions (C). (N = 3)

The number of mitochondria was evaluated by flow cytometry, by Mitotracker staining and by transmission electron microscopy TEM at P2, under hypoxia and normoxia in 3 independent experiments. Cells were analyzed by flow cytometry. The mean fluorescence intensity of hypoxic cells compared to normoxic cells was 47.7 versus 117 on histograms (Figure 3A). Thus hypoxia did inhibit the biosynthesis of mitochondria. The same cells were also permeabilized and stained with Mitotracker and analysed on fluorescence microscopy. As shown in Figure 3B, normoxic cells looked brighter than the hypoxic ones. We observed a 50 to 75% inhibition of mitochondrial biogenesis by counting the mitochondria on TEM sections. Strikingly, hypoxic cells looked less differentiated than normoxic ones. They displayed larger and less convoluted nuclei and more abundant nucleoli, and a higher nuclei/cytoplasm index, although the size of cells was very similar under both conditions (Figure 3C).

### Long term hypoxia stimulated the differentiation of MSC in adipocytes and osteocytes

**Figure 4:**
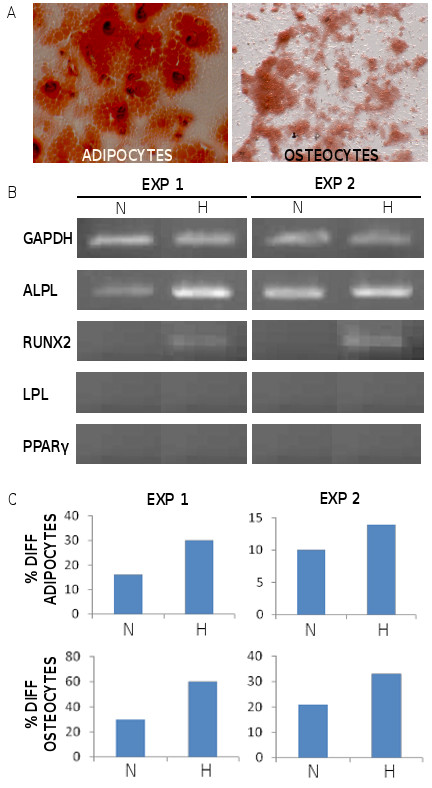
**Culture in hypoxia enhanced the potential of MSC to differentiate in adipocytes (A) and osteocytes (O)**. MSC were cultured in hypoxia or normoxia until P2, and shifted to adipocyte- or osteocyte-specific differentiation conditions respectively for 2-4 more weeks. The aspect of differentiated colonies under hypoxia is shown (A). In 2 independent experiments (B), MSC were harvested at P2, RNA from normoxic (N) or hypoxic (H) cells was extracted, reverse-transcribed and amplified by PCR with primers specific for control GAPDH and the indicated genes, representative of adipocytic (LPL, PPARγ) and osteocytic (ALPL, Runx2) lineages. The same cells were grown in osteocytic and adipocytic-specific medias and colonies were counted and compared to the numbers of CFU-F generated in MSC-specific medium (C).

MSC were grown from the start in normoxia versus hypoxia until P2, then washed and cultured in osteogenic or adipogenic lineage-specific media. The cells were kept in normoxia and hypoxia during the differentiation process. Differentiated cells were characterized by conventional histology staining (Figure 4A) and by RT-PCR for the amplification of lineage-specific transcripts for adipocytes (LPL, PPARγ) and osteocytes (ALPL, Runx2), respectively. The later transcripts were investigated in MSC at P2 before they were cultured in differentiating conditions. As shown from 2 independent experiments in Figure 4B, the expression of ALPL was stronger in hypoxic MSC than in normoxic cells. Further, while Runx2 transcription was undetectable in normoxic MSC, it was induced in hypoxic cells. This suggested that hypoxic cells were more prone to osteogenic differentiation than normoxic cells. We indeed observed that hypoxic MSC generated more (50% to 100% increase) osteogenic and adipogenic colonies, than normoxic MSC (Figure 4C).

**Figure 5:**
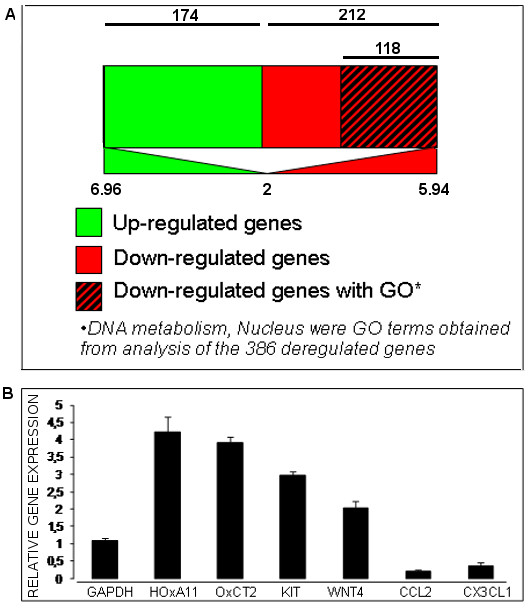
**Genetic reprogramming of MSC under hypoxia**. MSC from 3 donors were cultured until P0 and P2. RNA was extracted from each culture, processed and hybridized on Agilent DNA microarrays. Diagram A shows the GO analysis of differentially expressed transcripts at P0. Six transcripts were chosen and amplified by qPCR in order to validate gene array results obtained after long term hypoxia using the same extracts. Mean +/- SD from 3 experiments.

MSC from 3 distinct donors were cultured in normoxic and in hypoxic conditions. The cells from each donor/condition were harvested at P0 and P2. RNAs were extracted thereafter, processed and hybridization with microarrays was performed in 6 independent experiments. Comparative analysis of transcriptome from MSC cultured at P0 in hypoxia versus normoxia revealed 386 dysregulated genes (1% out of 41,000 genes), of which 174 were up regulated (45%) and 212 were down regulated (55%). GO analysis performed on the 386 dysregulated genes revealed an over-representation of genes (122 genes, p < 0.1) involved in DNA metabolism (cell cycle, replication, M-phase, spindle organization and biogenesis) and/or coding for nuclear proteins (chromosome, spindle, nucleus). Among these 122 genes, 118 (98%) had a 2-6 fold decreased expression with the range [2-5.94] corresponding to *NAV2 *and *RRM2 *genes respectively. Figure 5 and Table 1 summarize the analysis and showed the ten first down regulated genes after short culture in hypoxia. Contrary to down regulated genes, we showed no significant GO over-representation for up regulated genes. Nevertheless, we could observe a strong trend of over representation for genes coding for membrane receptors (CD93, ZP1, ESAM, protocadherin 17) and paracrine factors (Leptin, angiopoietin 2, VWF) with a range of over expression [2-6.96] corresponding to *ALDOC *and *TCF1 *genes respectively. Table 1 depicts the ten up regulated genes at P0 in hypoxia.

**Table 1:** Ten first down and upregulated genes at P0 in hypoxia.

Gene	Fold expression H/N	Genbank Accession	Putative Function
**Down Regulated**			
RRM2	5.94	NM_001034	Ribonucleotide Reductase
XRCC2	5.80	CR749256	X Ray damage DNA Repair
KIF24	5.16	AK001795	Kinesin: chromatid assembly
POLQ	4.99	AF090919	DNA polymerase theta
E2F8	4.95	NM_024680	Cell cycle progression
FANCD2	4.93	NM_001018115	DNA Repair
ESCO2	4.86	NM_001017420	Sister chromatid cohesion
AURKB	4.74	NM_004217	Chromosome segregation
CENPN	4.48	AK023669	Binding to Centromeres
MKI67	4.46	NM_002417	Cell proliferation
			
**Up Regulated**			
TCF1	6.96	NM_000545	Hepatic Transcription Factor
LEP	6.39	NM_000230	Metabolism, apoptosis, angiogenesis
ANGPT2	5.69	NM_001147	Antagonise vascular remodelling
ZP1	5.47	NM_207341	Sperm binding to zona pellucida
VWF	5.41	NM_000552	Platelet binding to endothelium
GIMAP4	5.37	NM_018326	T-cell development, Tumor suppressor ?
CD93	4.56	NM_012072	Intercellular adhesion, clearance apoptotic cells
PLVAP	4.25	NM_031310	Adhesion of Vascular Endothelial cells ?
ESAM	4.24	NM_138961	Adhesion of Endothelial cells
PCDH17	4.14	NM_001040429	Cell-cell connexions in the brain

Comparative analysis of transcriptome from MSC at P0 in hypoxia versus normoxia. Mean from 3 distinct samples.

Comparative analysis of transcriptome from MSC cultured at P2 in hypoxia versus normoxia revealed 519 dysregulated genes, of which 264 were up regulated (50.9%) and 255 were down regulated (49.1%). Gene ontology analysis performed on the 519 deregulated genes revealed an over-representation of genes involved in cell plasticity (48 genes, p < 0.1) and adhesion (37 genes, p < 0.1). When we increased the stringency of analysis by selecting genes that were 4-fold differentially expressed on 4 arrays, we eliminated genes for cell adhesion GO term but not for plasticity. All these 48 genes characterizing plasticity GO term were up regulated with a range between 4.2 and 58.3. Table 2 shows the ten first up regulated genes after long term culture (P2) in hypoxia. Several transcripts were validated by quantitative, real time PCR (qPCR) (Figure 5B). The results matched that of the gene arrays with enhanced expression of HOXA11, KIT, WNT4, OXCT2 and inhibited expression of CCL2, CX3CL1 under hypoxia.

**Table 2:** Ten first deregulated genes at P2 in hypoxia.

Gene	Fold expression H/N	Genbank Accession	Putative Function
SMOC2	58.32	NM_022138	Promotion of Matrix assembly
PLEKHA6	13.59	NM_014935	Adhesion
DMKN	10.79	NM_033317	Epithelial cell differentiation
KIT	8.62	NM_000222	Stem cell Proliferation
LAMA1	8.49	NM_005559	Development Retina and Myocytes
SNTG2	7.52	NM_018968	Eye development
GPR56	6.01	NM_201525	Neural development
OXCT2	5.81	NM_022120	Ketone body utilisation
NOG	5.13	NM_005450	Neural tube fusion, joint formation
HOXA11	4.80	NM_005523	Uterine development

Stimulation of plasticity genes under hypoxia according to GO analysis.

## Discussion

Hematopoietic and Stromal Stem Cells adapt themselves to hypoxia in culture which probably reflects their native hypoxic microenvironment [1-3]. Accordingly, several teams cultured HSC and MSC in hypoxic conditions in order to study their differentiation capacity [8-16,19]. Another goal of these experiments is the hope of expanding these cells while maintaining their "stemness" properties. Although data from various laboratories are difficult to compare due to wide variations in oxygen tension, ranging from 0.1 to 5%, and the duration of culture, ranging from a few hours to 2 months, a few studies evidenced an early growth inhibition under hypoxia [16]. Hypoxia induces cell cycle arrest in mammalian cells, however stem cells are more resistant to hypoxia than their progenies again reflecting their natural environment and their intrinsic quiescent state. We performed MSC cultures in 5% O_2 _which may be physiological for bone marrow stem cells [20]. As MSC and HSC form a single bone marrow niche [21], 5% O_2 _tension is likely to be physiological for MSC as well. We observed that MSC grew slower under 5% O_2 _than under 21% O_2 _until P1, and gained a progressive growth advantage in the next passages, which matched previously published results [16]. Meanwhile, hypoxic MSC expressed more adhesion and extracellular matrix molecules in early and late cultures, contained less mitochondria and displayed undifferentiated morphological features. In brief, early growth inhibition was somewhat expected and strikingly, GO analysis assigned down regulated genes to DNA metabolism and repair (POLQ, RRM2, XRCC2, FANCD2), cell cycle progression (E2F8, MKI67) and chromosomal organization (CENP-B, AURKB, KLF4) in agreement with our data on proliferation and colony size. Such inhibition likely contributes to the maintenance of MSC in a quiescent state, inasmuch as the inhibition of mitochondria may protect MSC from apoptosis. How could we reconcile these data with our observation that hypoxic MSC gained a growth advantage over normoxic MSC at late passages? The contradiction may be apparent. One possibility is that these cells became more sensitive to growth factors present in the serum. Whether growth advantage is due to a stimulation of proliferation pathways or to the expression of receptors for cytokines and growth factors or both, is worth investigating. Note in this respect that CXCR4 was induced by hypoxia.

As MSC in their niche are supposed to be quiescent and multipotent, these properties are apparently dissociated in our *in vitro *model, with quiescence being observed at early passages, whereas multipotency is augmented at late passages. Until we understand the *in vivo *signature of MSC, we cannot draw conclusions and pretend that *in vitro *culture in hypoxia mimics the niche.

Although expected from previous studies and suggested by our morphological observations, maintenance of stem cell characteristics at early passages under hypoxia was not inferred from GO analysis. Early induced genes were not assigned to multipotency but instead belonged mostly to adhesion molecules such as Von Willebrand Endothelial Cell Adhesion molecule and Protocadherin (Table 1). However, several genes may clearly affect stemness. CD93 regulates the clearance of apoptotic cells, a function critical to development, maintenance of homeostasis and tissue repair [22]. The WNT-related transcription factor TCF1 may regulate MSC and enhance their osteogenic differentiation [23]. At variance with the above genes, 8 genes potentially involved in the control of differentiation towards adipocytes, osteocytes and chondrocytes [1] were not modified by hypoxia [Additional file 1]. Strong expression of adhesion molecules may be physiologically relevant and correlate with broader differentiation potential of hypoxic MSC. Indeed VWF is a marker of endothelial commitment [24] and PLVAP, reported here for the first time in MSC is a leukocyte trafficking molecule [25] which may help transendothelial migration of MSC from the bone marrow. Stimulation of Leptin is also meaningful as a recent work demonstrated that it helps maintain mesenchymal progenitor cells undifferentiated [26]. This result also shows that hypoxia impacts the metabolism of MSC in agreement with a study on rat MSC [27]. In this study however, the duration of hypoxia was 24 hours only. Yet, several genes involved in adhesion and extracellular matrix were stimulated.

Hypoxia generates "plasticity". At P2 in hypoxia, only one group of genes was stimulated and was assigned to plasticity. SMOC2 is the first induced gene (Table 2) and plays a role in angiogenesis and extracellular matrix assembly [28], yet a recent article demonstrated that a related protein increases life span and fecundity in Drosophila [29]. Kit gene was induced thus correlating with proliferation [30]. LAMA1/laminin [31] and SNTG2/syntrophin gamma-2 [32,33] are both involved in retinal and eye development whereas GPR56, a seven-transmembrane domain protein, is involved in brain cortical patterning [34].

We have observed that hypoxia stimulated several genes which converge to maintain the cells in an undifferentiated state, and facilitate transendothelial migration of MSC (Table 1 and 2). In parallel, hypoxia inhibited the expression of genes involved in cell proliferation (Table 1). This transcription profile probably reflects the intrinsic genetic program of MSC *in vivo *as these cells are quiescent, and endowed with migration and multilineage differentiation capacities. With respect to migration, note that CXCR4 was induced by hypoxia [Additional file 1 and reference 3] with potential implications in the egress of MSC from the bone marrow. This is in contrast with the cell surface phenotype of MSC which was almost unaffected in our experiments and in others [17]. Note however that STRO-1 was expressed only transiently in cultured hypoxic but not in normoxic cells. This is not totally surprising since STRO-1 expression is gradually lost during culture expansion [18,35]. Even though STRO-1 is useful to isolate MSC from various tissues, it is not positive on all MSC [36]. Interestingly, STRO-1+ cells displayed enhanced expansion and multilineage differentiation potentialities [37,38]. Thus, the expression of STRO-1 on hypoxic MSC may not be fortuitous and reflects multipotential status.

Our results may have physiological & medical applications. Oxygen tension is a critical parameter, possibly the most important one, in the culture of stem cells. As nestin-positive MSC and HSC form a unique bone marrow niche [21], hypoxia is undoubtedly a physiological milieu for MSC. In this respect it is worth mentioning that nestin was induced by hypoxia in our experiments [Additional file 1]. Given the ever growing therapeutic applications of MSC in regenerative medicine [39] and in autoimmune diseases [40], the impact of O_2 _on the functions of MSC should be carefully evaluated. For instance, intravenous injection of MSC results in their accumulation in the pulmonary parenchyma. Although this was sufficient to treat experimental septic shock [41], dissemination of MSC into other organs may be necessary to treat systemic diseases; induction of molecules involved in transendothelial migration as observed in our experiments may be helpful in this setting. Conversely however, hypoxia may be detrimental to other purposes. MSC inhibit TH17 cells in a CCL2-dependent manner by processing this chemokine to an antagonistic derivative, and may be helpful in the treatment of Experimental Allergic Enkephalitis (EAE) [42]. Note in this respect that the transcription of CCL2 in MSC was inhibited under hypoxia in our experiments. Altogether our data demonstrate that hypoxia favoured the "undifferentiation program" of MSC, it remains to evaluate the impact of hypoxia on each desired function of these cells in the event of medical applications.

As the Holy Grail is to use tissue-specific cells derived from MSC in regenerative medicine, culture of MSC in hypoxia at least until P2 in order to induce the expression of a broad range of tissue-specific genes, may be beneficial, inasmuch as it also enhanced the cell numbers in parallel to their differentiating capacity. In this respect differentiation experiments should be carried to evaluate the potential of MSC to generate endothelial cells, myocytes and neurons. Finally, the most relevant result here is the demonstration of induction of plasticity, a major property of MSC, at variance with HSC [43].

## Conclusions

The duration of hypoxemia is a critical parameter for the differentiation capacity of MSC. Hypoxia maintains the cells undifferentiated and in parallel enhances the expression of genes involved in the development of various, mesodermal and non mesodermal, cell lineages. In this respect hypoxia may increase both the multipotency and the transdifferentiation potential of MSC.

## Methods

### Isolation and culture of human MSC

MSC were obtained from bone marrow samples from 6 adult donors with their informed consent following the bylaws of the ethical committee of the Nancy University. MNC were counted and plated at 50 × 10^3 ^cells/cm^2 ^and cultured in Minimal Eagle Medium (α-MEM; Cambrex) supplemented with 10% fetal bovine serum, glutamine 2 mM and penicilin. They were incubated at 37°C under an atmosphere of 5% CO_2 _in either 21% O_2 _(herein referred to as normoxia) or 5% O_2 _(hypoxia). Hypoxia was maintained in a dedicated incubator (Sanyo) connected to CO_2 _and N_2 _injectors, in which relative N_2 _was increased to reach the desired O_2 _concentration. Medium was changed twice weekly. MSC were isolated by adherence to plastic. In primoculture, cells were harvested after 21 days (passage 0 or P0) and counted by trypan blue (Sigma-Aldrich). For the next passages (P1, P2 or P3), cells were subcultured at different seeding densities (100 or 1000 cells/cm^2^) for 14 days, trypsinized and counted.

For colony-forming unit fibroblast (CFU-F) assays, 1000 and 10000 MNC from total BM were seeded in 60 cm^2 ^dishes in duplicate. They were cultured for 14 days in normoxic and hypoxic conditions. After that, cells were washed 3 times with PBS and stained with Cristal Violet solution (Sigma-Aldrich). Plates were scanned and CFU-F of more than 30 cells, were scored. The size of the colonies was determinates thereafter using the "Image J" software. CFU-Fs were counted at P0, P1, P2 and P3.

To determine the population doubling (PD), cells in P1 and P2 were seeded at 100 or 1000 cells/cm^2 ^in T75 flasks and trypsinized after 14 days. Cells were counted and population doubling calculated as: PD = log (N_f_/N_i_)/log 2, N_f _= Final cell number; N_i _= Initial cell number.

### Microscopy

For electron microscopy, cells were either trypsinized and pelleted before processing or processed as cell monolayers in 12 well plates. Briefly, cells were fixed for 2 h at 4°C in 2.5% glutaraldehyde containing 0.1 M Na cacodylate, then rinsed for 3 h in cacodylate buffer and incubated for 30 min at RT in 1% osmium tetroxyde in cacodylate buffer, rinsed and dehydrated in increasing concentrations (30, 50, 70, 80, 90%) of ethanol, for 5 min each, then in 100% ethanol for 3 × 20 min. Finally the cells were embedded in a 50/50 volume mixture of resin and propylene oxide. A volume of 30 ml of resin EMS (Euromedex, France) is made by mixing 18.2 ml of EMBED (spi-pon 812), 12.4 ml DDSA, 9.4 ml NMA, and 0.7 ml DMP30 for 20 min RT on a stirring magnet. Cell monolayers on plastic wells were treated twice with 100% xylene and semi thin (1.5 mm) or ultra thin sections (70-90 nm) were performed using an ultra microtome (Reichert-Yung). Sections were observed on a Phillips CM12 electron microscope and photographed.

For optical microscopy and mitochondrial staining, cells were incubated with 100 nM Mitotracker orange CMTMRos (Invitrogen), for 45 min at 37°C, washed in 1× PBS, and photographed on an Olympus DP-70 microscope.

### Flow Cytometry

For mitochondrial staining, cells were incubated as above, enzymatically detached and resuspended in phenol-red free medium before flow cytometry analysis.

For surface antigen expression on culture-expanded MSC, cells were detached, washed, pelleted and resuspended in DMEM medium without phenol red and incubated for 20 minutes at room temperature with antibodies in a final volume of 100 μl and eventually resuspended in 4% paraformaldehyde until analysis on FC500 Beckman Coulter flow cytometer. We used monoclonal antibodies listed in Table 3. Antibodies were conjugated to fluorescein isothiocyanate (FITC), allophycocyanin (APC) or phycoerythrin (PE). Each sample was stained with either CD34 or CD45 (negatively) and with one of the other MAbs from Becton Dickinson (BD, USA), Beckman Coulter (BC, Canada) or Santa Cruz (SC, USA).

**Table 3:** List of monoclonal antibodies

Antibody	Conjugated	Isotype	Reference
Anti-CD34	FITC	IgG1 Mouse	IM1870, BC

Anti-CD45	FITC	IgG1 Mouse	A07782, BC

Anti-CD90	PE	IgG1 Mouse	IM3600U, BC

Anti-CD105	PE	IgG3,k Mouse	A07414, BC

Anti-CD271	PE	IgG1, k Mouse	557196, BD

Anti-CD106	PE	IgG1, k Mouse	555647, BD

Anti- CD166	PE	IgG1, k Mouse	559263, BD

Anti-CD73	PE	IgG1, k Mouse	550257, BD

Anti-CD29	APC	IgG1, k Mouse	559883, BD

Anti-CD44	APC	IgG2b, k Mouse	559942, BD

Anti-STRO-1	PE	IgM Mouse	sc-47733, SC

Isotype control	APC	IgG2b, k Mouse	555745, BD

Isotype control	PE	IgG1, k Mouse	555749, BD

Isotype control	PE	IgM Mouse	sc-2870, SC

Isotype control	APC	IgG1, k Mouse	555751, BD

Isotype control	FITC	IgG1, Mouse	A07795, BC

Isotype control	PE	IgG1, Mouse	A07796, BC

### MSC Differentiation Assays

The potential of MSC to differentiate into the adipogenic and osteogenic lineages was verified. MSC were enzymatically detached from the culture flasks at nearly confluence and replated in 60 cm^2 ^dishes at different densities and with specialized culture mediums according to the desired differentiation:

#### Adipogenic differentiation

MSC cells were seeded at 500 cells/cm^2 ^and cultured for 14 days with standard culture medium. After that we induced differentiation by supplementing standard culture medium with dexamethasone 1 μM, indomethacin 60 μM and insulin 5 μg/ml for 21 days. Cells were then washed with PBS, fixed in 10% formaldehyde, washed with 60% isopropanol and stained with Oil red O Solution (Sigma-Aldrich) to detect lipid droplets within the cells.

#### Osteogenic differentiation

MSC cells were seeded at 100 cells/cm^2 ^and cultured for 14 days with standard culture medium. After that we induced differentiation by supplementing standard culture medium with ascorbic acid 60 μM, β-glycerol phosphate 10 mM and dexamethasone 0.1 μM for 21 days. Cells were washed with PBS and fixed in ice-cold 70% ethanol and stained with Alizarin Red S (pH: 4.1; Sigma-Aldrich) to detect Ca^2+ ^deposits.

Differentiation was further assessed by PCR amplification of lineage-specific transcripts and GAPDH as control using primers listed in Table 4.

**Table 4:** List of primers for amplification of lineage-specifics transcripts (GAPDH is used as control)

Gene Product	Primers	Product Size (bp)
GAPDH (NM_002046.3)	Fw: 5'-AATCCCATCACCATCTTCCAGG-3' Rv: 5'-AGAGGCAGGGATGATGTTCTGG-3'	417
ALPL (NM_000478)	Fw: 5'-CTGGACCTCGTTGACACCTG-3' Rv: 5'-GCGGTGAACGAGAGAATGTC-3'	546
LPL (NM_000237.2)	Fw: 5'-AAAGCCCTGCTCGTGCTGAC-3' Rv: 5'-ACAGGATGTGGCCCGGTTTA-3'	406
PPARG (NM_005037.5)	Fw: 5'-GGAGAAGCTGTTGGCGGAGA-3' Rv: 5'-CACAATGCTGGCCTCCTTGA-3'	431
RUNX-2 (NM_001015051.2)	Fw: 5'-AACTTCCTGTGCTCGGTGCTG-3' Rv: 5'-GGGGAGGATTTGTGAAGACGG-3'	268

### Lineage specific transcript analyses

#### Microarrays

Total RNA was extracted and purified from MSC treated in normoxic or hypoxic conditions (P0-P2) according to the RNeasy Mini Kit protocol (Qiagen, Valencia, CA, USA). To perform whole Human Genome Oligo (60-mer) array gene expression analysis, total RNA was extracted from MSC treated on normoxic or hypoxic condition (n = 4) each, including technological and biological replicates). For each sample, 350 ng of total RNA was reverse transcribed, linear amplified, and labelled with Cy3 (one colour protocol) using Agilent's Low RNA Input Linear Amplification Kit PLUS, according to manufacturer's instructions. After labelling, samples were measured on a Nanodrop microarray module for labelling efficiency and quantification. Samples were then hybridized on Agilent 4 × 44 K whole human genome GE arrays (Agilent Design #014850) at 65°C for 17 h. After washing in GE washing buffers, the slide was scanned with Agilent Microarray Scanner G2565CA. Feature extraction software (Version 9.5.3.1, Agilent technologies Inc., CA, USA) was used to convert the image into gene expression data. Genespring GX10 software (Agilent technologies Inc., CA, USA) was used to compile and analyse data. First normalized data (background substracted) were filtered on expression (lower and upper cut-off 20 and 100 respectively for 100% of signal), then on error (CV < 50% for 100% of signal). Only genes that were 2-fold differentially expressed on 4 arrays were scored as significant and used for analysis. Biological process and cellular component of genes were classified according to Gene Ontology (p < 0.1).

#### Real time PCR

For quantitative PCR, the cDNA used for DNA chip analysis were amplified using the primers listed in Table 5.

**Table 5:** List of primers used for qPCR (GAPDH is used as a calibrator)

Gene Product	Primers	Product Size (bp)
HoxA11 (NM_005523.5)	Fw: 5'-TTGAGCATGCGGGACAGTT-3' Rv: 5'-GTACCAGATCCGAGAGCTGGAA-3'	87
OxCT2 (NM_022120.1)	Fw: 5'-GAGTTCAACGGCGACCACTT-3' Rv:5'-GCGCTTCTCCTGAAGACCA-3'	110
V-KIT (NM_000222.2)	Fw: 5'-GGCGACGAGATTAGGCTGTT-3' Rv: 5'-CATTCGTTTCATCCAGGATCTCA-3'	77
CCL2 (NM_002982.3)	Fw: 5'-ACTCTCGCCTCCAGCATGAA-3' Rv: 5'-GGGAATGAAGGTGGCTGCTA-3'	72
CX3CL1 (NM_002996.3)	Fw: 5'-TGACATCAAAGATACCTGTAGC-3' Rv: 5'-CTCGTCTCCAAGATGATTGC-3'	88
WNT4 (NM_030761.4)	Fw: 5'-AGCAACTGGCTGTACCTG-3' Rv: 5'-CTGGATCAGGCCCTTGAG-3'	87
GAPDH (NM_002046.3)	Fw: 5'-CGCTCTCTGCTCCTCCTGTT-3' Rv: 5'-CCATGGTGTCTGAGCGATGT-3'	81

The reactions were carried out in 25 μL volume containing cDNA and Master mix (Power SYBR Green PCR Master Mix kit). Thermocycling conditions were 40 cycles of two steps: 15 sec at 95°C plus 1 min at 60°C. Detection was performed using a Mastercycler^® ^ep *realplex *real-time PCR system (Eppendorf). The relative RNA level and fold change in hypoxia/normoxia condition were calculated using the 2^-ΔCt ^using GAPDH as a calibrator.

### Statistics

All statistics were carried using the bilateral Student's t test on Excel program, in order to compare the data in normoxia versus hypoxia.

## Authors' contributions

LB did the cultures and PCRs and participated in Microscopy and FACS analysis, CN did the gene arrays, BF did the microscopy, N de I did the cultures, M de C helped in FACS, NT participated in the experiments and culture, AD designed the experiments, wrote the article and participated in microscopy, gene array and FACS analysis.

All authors read and approved the final manuscript.

## Supplementary Material

Additional file 1 **HN-fold change 4**. Click here for file
